# Comparison of response to LPS-induced sepsis in three DBA/2 stocks derived from different sources

**DOI:** 10.1186/s42826-020-00079-5

**Published:** 2021-01-07

**Authors:** Ji Won Park, Su Jin Lee, Ji Eun Kim, Mi Ju Kang, Su Ji Bae, Yun Ju Choi, Jeong Eun Gong, Kil Soo Kim, Young-Suk Jung, Joon-Yong Cho, Yeon Shik Choi, Dae Youn Hwang, Hyun Keun Song

**Affiliations:** 1 Department of Biomaterials Science, College of Natural Resources & Life Science/Life and Industry Convergence Research Institute/Laboratory Animals Resources Center, Pusan National University, Miryang, South Korea; 2 College of Veterinary Medicine, Kyungpook National University, Daegu, South Korea; 3 College of Pharmacy, Pusan National University, Busan, South Korea; 4 Exercise Biochemistry Laboratory, Korea National Sport University, Seoul, South Korea; 5 Department of Biomedical Analysis, Korea Bio Polytechnic College, Nonsan, South Korea; 6 Central Research Institute, Kinesiences Co., Seoul, South Korea

**Keywords:** DBA/2, DBA/2Kor, LPS, Sepsis, Pro-inflammatory cytokine

## Abstract

Sepsis, one of the most fatal diseases in the world, is known to culminate in multiple organ failure due to an uncontrolled inflammatory response. Hence, the use of animal models in sepsis research is very important to study complex immune responses. The current study was undertaken to compare commercial stocks with KFDA stocks of DBA/2 mice as an animal model for sepsis study. To compare responses of DBA/2 mice to lipopolysaccharides (LPS)-induced sepsis, we measured altered characteristics of various factors associated with sepsis, including survival curves, organ failure and inflammatory response, in DBA/2Korl stock and two commercial stocks (DBA/2A and DBA/2B). Survival rates after LPS exposure were similar for DBA/2Korl and DBA/2B; however, for times over 20 h, survival rates were reduced and concentration dependent in DBA/2A. In order to evaluate multiple organ failure caused by sepsis, H&E stains were evaluated for liver and spleen tissues obtained in the early (2 h) and later (20 h) stages after exposure to LPS; no significant differences were observed between the three stocks. mRNA and protein levels of proinflammatory cytokines were assessed for evaluating inflammatory reactions, and were found to increase in a dose-dependent manner in most DBA/2 mice after LPS treatment. However, no changes were observed in the mRNA levels of proinflammatory cytokines at 20 h after LPS exposure in the DBA/2A stock. The induction of inflammation-mediated factors by LPS exposure did not induce alterations in the mRNA levels of COX-2 and iNOS in all three DBA/2 stocks. Our results indicate that response of DBA/2Korl to LPS-induced sepsis is similar to the two commercial DBA/2 stocks, thus representing its potential as a useful biological resource established in Korea.

## Introduction

Sepsis is defined as an infection as well as inflammatory reactions that result in tissue damage [1]. It is globally accepted as one of the deadliest diseases, having higher mortality rates than seizures or strokes. The United States has reported an occurrence of sepsis in 3 per 1000 people, and is considered as the most common cause of death in the intensive care units, except for patients with cardiovascular disease [2, 3]. Moreover, some reports state that the death rate of sepsis patients is around 30%, and that 85% of sepsis patients do not receive proper treatment [4–7]. Despite these high mortality rates, the pathophysiological mechanisms for progression and treatment of sepsis are not fully understood [8–11].

In general, sepsis is recognized to be caused by an uncontrolled inflammatory reaction, usually initiated by microbial agents such as LPS (the outer membrane component of gram-negative bacteria), causing fatal harm to the body, as the excessive immune response is systemic [12]. Reactive treatment strategies, such as antibiotics for infections, have been developed, but new treatment methods and screening of drug candidates are imperative for the prevention and treatment of sepsis. Hence, the use of animal models as alternatives is required as it is impossible to test new treatments or pursue pathological knowledge having potential for human patients.

Recently, the mouse model of sepsis has been in the spotlight for its ease of experimentation, low cost, and ease of genetic modification despite the differences in genomes between mice and humans, and some septic pathogenesis [13–15]. While there are no murine models that fully reflect all factors associated with sepsis due to the aforementioned differences in the genomes of humans and murine, the various murine models available can represent the requirements according to the focus of the study.

Among the mouse sepsis models, model classification by disease source can be largely categorized into three types: 1) endotoxin administration (LPS), 2) administration of pathogens (*Escherichia coli*), and 3) endogenous barrier decay (vascular fixation and punk model (Cecum Ligation and Puncture, CLP) [16]. In particular, LPS induced sepsis animal models have high levels of circulating inflammatory cytokines (TNF-α, IL-1β and IL-6) and interferon-γ immediately after administration of LPS [17–19].

DBA/2 mice are relatively early isolated inbred mice used for diseases such as toxicity or safety, immunology, or auditory seizure studies. In 2001, Lorenz reported that embedded mouse species, especially C57BL/6 mice, were more responsive to the LPS than DBA/2 mice, and that the Th2 type immune response was better than TH1 [20–22]. Therefore, DBA/2 mice are considered a useful strain for LPS-induced sepsis research.

The current study compares the LPS-induced sepsis between the DBA/2Korl stock and two commercial DBA/2 stocks, to verify the characteristics of the DBA/2Korl mice as established by the Korea FDA. Our study results provide numerous scientific evidence of similar responses to LPS-induced sepsis, as observed in the immune system of DBA/2Korl, DBA/2A and DBA/2B stocks.

## Materials and methods

### Design of animal experiment

All animal protocols used in this study were reviewed and approved by the Pusan National University-Institutional Animal Care and Use Committee (PNU-IACUC, approval number PNU-2019-2202). Male DBA/2 mice (7-weeks-old) were obtained from three different sources. The DBA/2Korl mice were kindly provided by the Department of Laboratory Animal Resources of the National Institute of Food and Drug Safety Evaluation (NIFDS, Chungju, Korea). The other two stocks of DBA/2 mice (DBA/2A and DBA/2B) were purchased from vendors located in the United States (Vendor A) and Japan (Vendor B), respectively. All DBA/2 mice were maintained and treated at the Animal Resource Center of Pusan National University, which is certified by the Food and Drug Administration (FDA) (Accredited unit number; 000231), and Association for Assessment and Accreditation of Laboratory Animal Care (AAALAC) International, according to the National Institutes of Health guidelines (Accredited Unit Number; 001525). During the experiment, all mice were maintained in a specific pathogen-free (SPF) state under a strict light cycle (lights on at 08:00 h and off at 20:00 h), at 23 ± 2°C and 50 ± 10% relative humidity. Animals were provided *ad libitum* access to a standard irradiated chow diet (Samtako Biotech Inc., Osan, Korea).

LPS induced sepsis was stimulated as previously described [23]. Briefly, 8-week-old DBA/2 mice of each stock (*n* = 40) were assigned to either a non-impairment group (Vehicle treated group, *n* = 10) or impairment group (*n* = 30). The impairment group was further divided into Low LPS-treated (2 mg/kg, n = 10), Mid LPS-treated (5 mg/kg, n = 10), and Hi LPS-treated (10 mg/kg, n = 10) groups. Appropriate amount of LPS (Sigma-Aldrich Co., St. Louis, MO, USA) was intraperitoneally injected into each impairment group to induce experimental conditions. Survival of the mouse was observed at 2 h and 20 h after LPS administration. Thereafter, all animals were euthanized using a chamber filled with CO_2_ gas. Tissues and blood samples of all mice were subsequently harvested and subjected to experimental analysis.

### Histological analysis

Liver and spleen tissues were excised from the LPS-treated mice, fixed in 10% formalin, embedded in paraffin wax, processed routinely, and sectioned into 4 μm thick slices. These sections were subsequently stained with hematoxylin and eosin (H&E, Sigma-Aldrich Co.), and histopathological features were then examined by the Leica Application Suite (Leica Microsystems, Heerbrugg, Switzerland).

### Reverse transcriptase-polymerase chain reaction (RT-PCR)

The mRNA expressions of TNF-α, IL-6, COX-2 and iNOS were measured using RT-PCR. Total RNA was extracted from the spleen tissue of all experimental animals, with RNAzol CS104 (Tel-Test Inc., Friendswood, TX, USA). The isolated mRNA was reverse transcribed using M-MLV reverse transcriptase (Promega, Madison, WI, USA) at 42°C for 1 h, according to the manufacturer’s protocol, followed by addition of 10 pmol of the sense and antisense primers. The reaction mixture was then subjected to 28–32 cycles of amplification, conducted in a Perkin-Elmer Thermal Cycler using the following cycles: 30 s at 94°C, 30 s at 62°C, and 45 s at 72°C. The primer sequences used for target gene expression identification were as follows: TNF-α, sense primer: 5′- GGC CTC TCT ACC TTG TTG CC − 3′, anti-sense primer: 5′- CAG CCT GGT CAC CAA ATC AG -3′; IL-6, sense primer: 5′- TTG CCT TCT TGG GAC TGA TG − 3′, anti-sense primer: 5′- CCA CGA TTT CCC AGA GAA CA -3′; COX-2, sense primer: 5′-CAGGT CATTG GTGGA GAGGT GTATC-3′, anti-sense primer: 5′-CCAGG AGGAT GGAGT TGTTG TAGAG-3′; iNOS, sense primer: 5′- CGA AAC GCT TCA CTT CCA A − 3′, anti-sense primer: 5′- TGA GCC TAT ATT GCT GTG GCT − 3′; β-actin, sense primer: 5′- CAG GTC ATT GGT GGA GAG GTG TAT C − 3′, anti-sense primer: 5′- CCA GGA GGA TGG AGT TAT TAT AGA G − 3′. The experiment was repeated three times, and all samples were analyzed in triplicate. The final PCR products were separated on 1–2% agarose gel, followed by visualization after ethidium bromide staining. The density of a specific band was quantified using the Kodak Electrophoresis Documentation and Analysis System 120 (Eastman Kodak, Rochester, NY, USA).

### Enzyme-linked immunosorbent assay (ELISA) for TNF-α and IL-6 cytokine

After 20 h, all mice were fasted for 8 h, following which anesthesia was induced by intraperitoneal injection of Alfaxan (JUROX Pty Limited, Rutherford, Australia, 13 mg/kg body weight i.v.). Blood was subsequently collected from the abdominal veins using a 1 ml syringe attached to a needle (21 SWG), and centrifuged at 2,000 xg for 20 min at 4°C. Serum was collected and stored at 70°C before subjecting to further analysis. TNF-α and IL-6 concentrations in serum were quantified using a mouse TNF-α ELISA kit (Biolegend, San Diego, CA, USA) and interleukin (IL)-6 ELISA kit (Biolegend), respectively, according to the manufacturer’s instructions.

### Statistical analysis

Statistical analyses were performed with SPSS for Windows, release 10.10, standard version (SPSS, Inc., Chicago, IL, USA). One-way analysis of variance followed by Tukey’s post hoc test for multiple comparisons was performed to identify significant differences between groups. All values are reported as the mean ± S.E.M., and a *P* value < 0.05 is considered as significant.

## Results

### Response to LPS-induced sepsis in three different DBA/2 stocks

**Fig. 1:**
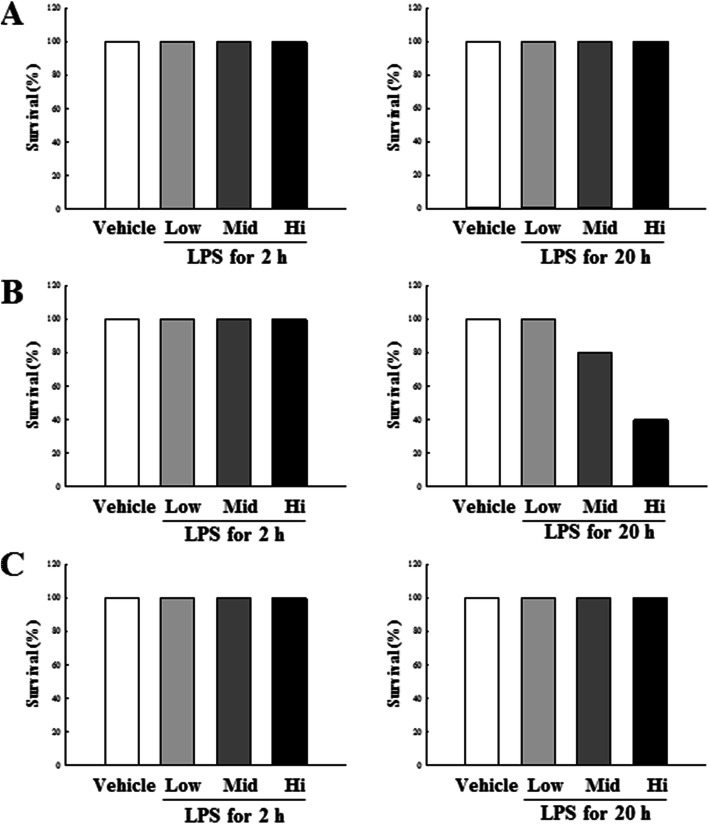
Comparison of the three DBA/2 stocks mortality in LPS-induced sepsis. The mortality was evaluated as the time of death of the three mice stocks: DBA/2Korl (**a**), DBA/2A (**b**), and DBA/2B (**c**), after exposure to indicated concentration of LPS or Vehicle. Left panel presents survival rate at the early time point (2 h) after exposure. Right panel presents survival rate at a late time point (20 h) after exposure

**Fig. 2:**
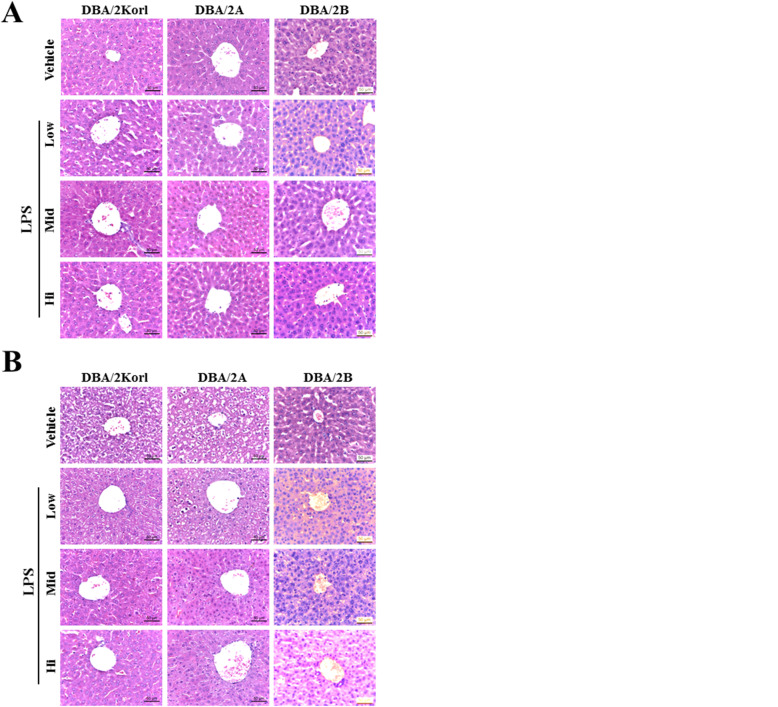
Determination of LPS-induced septic organ malfunctions among the three DBA/2 stocks, using Hematoxylin and Eosin (H&E) staining. Representative images show staining of H&E in liver (**a** and **b**) and spleen (**c** and **d**) tissues treated with Vehicle or LPS. **a** and **c** panel display tissues harvested at early time (2 h) from DBA/2Korl, DBA/2A, and DBA/2B stocks; **b** and **d** panel display tissues harvested at late time (20 h) from DBA/2Korl, DBA/2A, and DBA/2B stocks

To compare the LPS-induced sepsis reactions in three DBA/2 stocks, mice were administered three different concentrations of LPS, and survival was measured at 2 h and 20 h post exposure (Fig. 1). The DBA/2A stock was observed to be more sensitive to LPS exposure, as compared to the other two stocks. Moreover, increased mortality rate was observed at 20 h with increasing LPS concentrations. It is well documented that generally, in septic murine animal models, the animals die at early time point after the LPS inoculation, due to multiple organ failure induced by sepsis related to a severe inflammatory response. Therefore, in the current study, LPS was administered to compare the difference in sepsis reactions induced by LPS between stocks, and liver and spleen tissues were harvested at 2 h and 20 h to observe the presence of tissue damage. The H&E staining results of sample tissues obtained from each mouse stock at 2 h after LPS administration showed normal liver (Fig. 2a) and spleen (Fig. 2c) at all concentrations. These results are consistent with prior reports that survival rate 2 h after the LPS administration remains unaffected (Fig. 1). As presented in Fig. 2b and c, the liver and spleen tissues harvested 20 h after LPS inoculation shows normal tissue appearance in H&E staining results, with no differences observed between stocks.

### Alteration of septic inflammation response in LPS-treated DBA/2 stocks

**Fig. 3:**
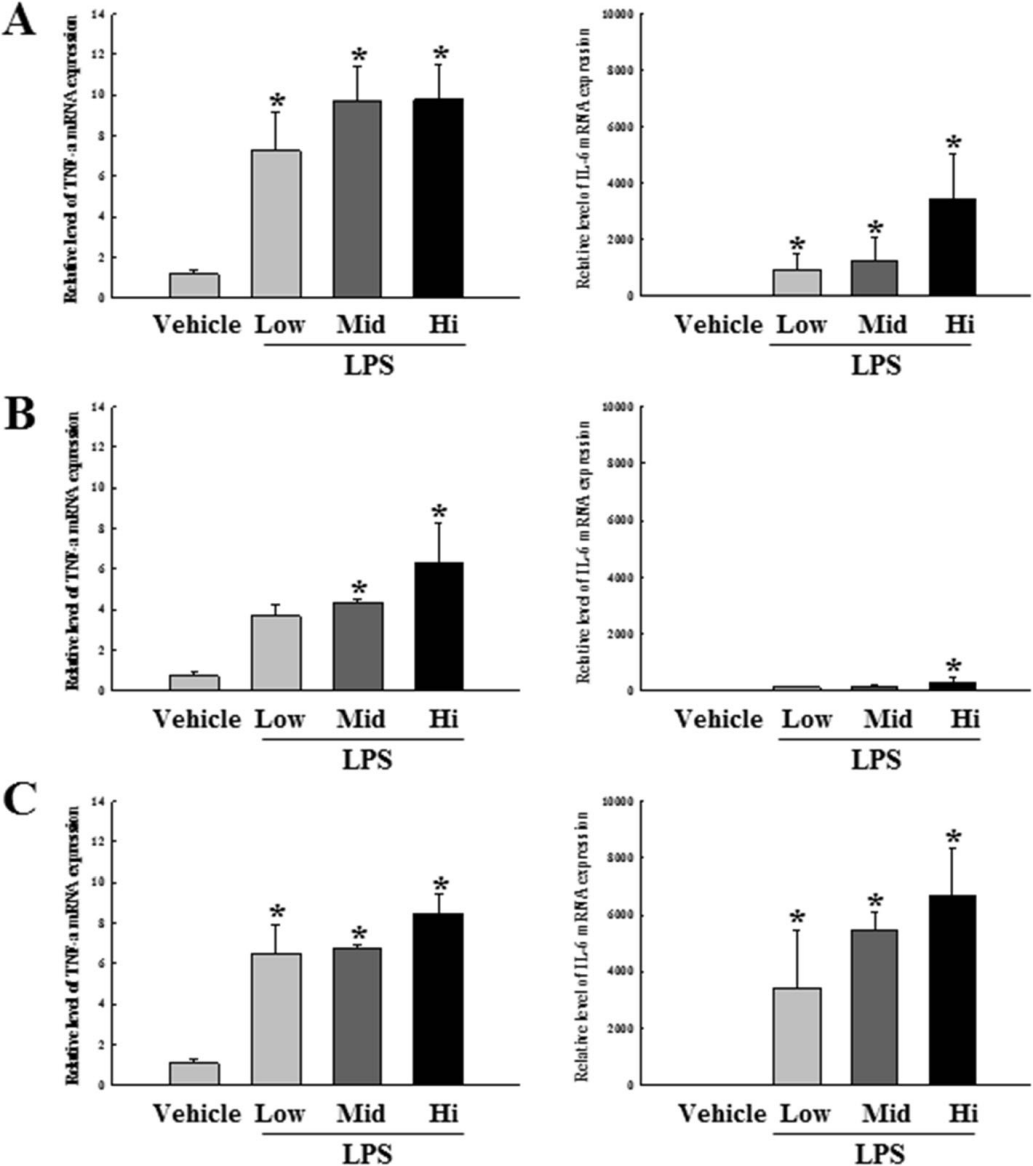
Comparison of cytokine expressions in spleen tissue of LPS or Vehicle treated DBA/2 stocks. Spleen tissue samples harvested at early time (2 h). The mRNA levels of cytokine (TNF-α and IL-6) were measured by RT-PCR using specific primers. Amplified PCR products were electrophoresed on agarose gel, and intensity of the specific bands was recorded by a gel documentation system. Each panel represents the mRNA expression level of inflammation related genes in DBA/2Korl (**a**), DBA/2A (**b**), and DBA/2B (**c**) mice after treatment with LPS or Vehicle. *P < 0.05 compared to the Vehicle treated group

**Fig. 4:**
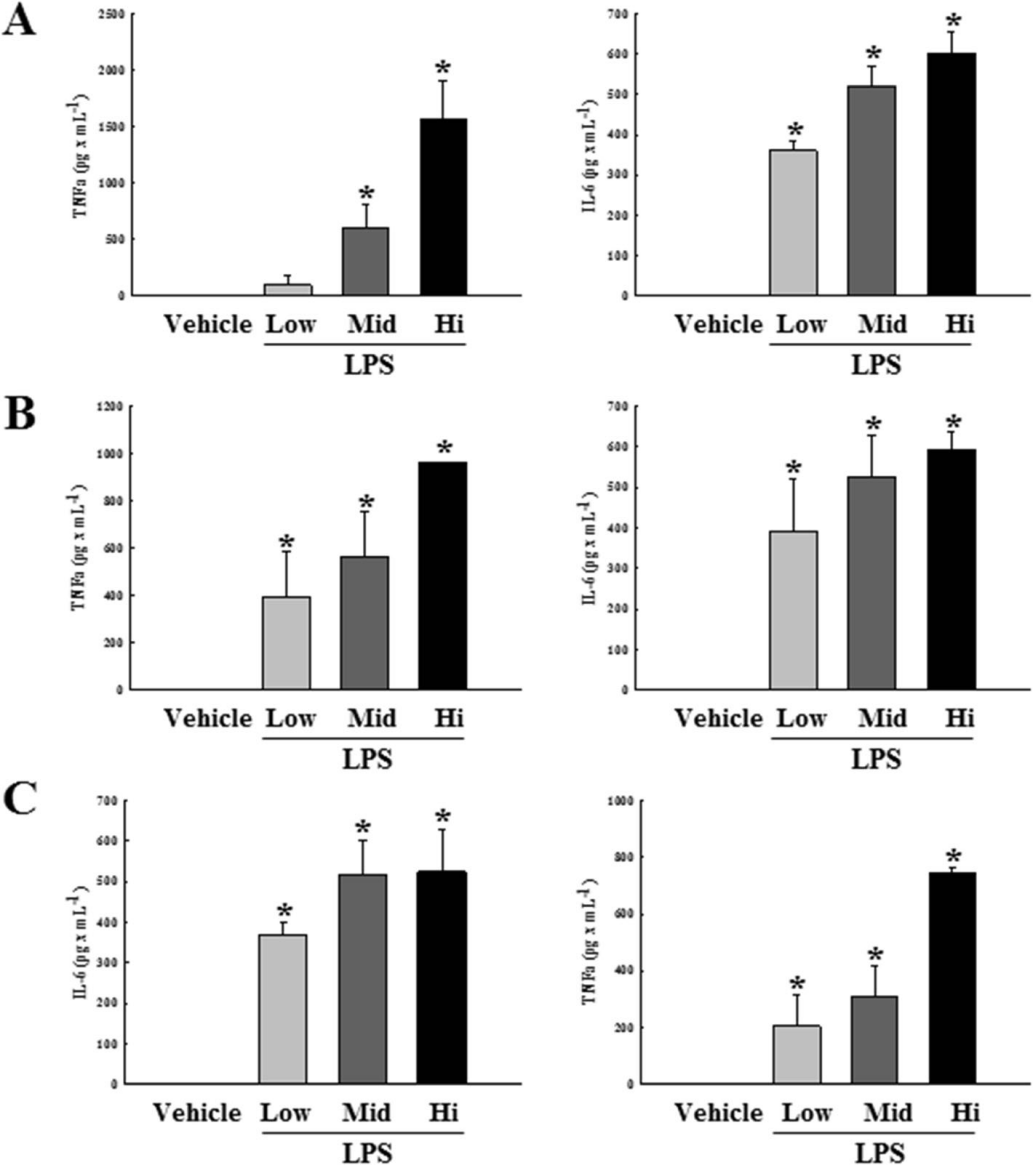
Comparison of cytokine production in blood samples of LPS or Vehicle treated three DBA/2 stocks. Serum samples were harvested at late time point (20 h). The protein levels of cytokine (TNF-α and IL-6) were measured by ELISA. The protein levels of cytokine (TNF-α and IL-6) were evaluated among DBA/2Korl (**a**), DBA/2A (**b**), and DBA/2B (**c**) mice after exposure to indicated concentration of LPS or Vehicle. Left panel present protein level of TNF-α after exposure to indicated concentration of LPS or Vehicle. Right panel present protein level of IL-6 after exposure to indicated concentration of LPS or Vehicle. Data represents the mean ± SD of n=8/group. *P<0.05 compared to the Vehicle treated group

To compare the LPS-induced septic inflammatory response in the three DBA/2 stocks, we measured the mRNA levels of pro-inflammatory cytokines TNF-α and IL-6, after exposure to varying doses of LPS. Dose-dependent increased expressions of the inflammatory cytokine mRNAs were observed in all LPS-treated groups, as compared to the Vehicle group. These patterns were commonly observed in all three stocks for TNF-α mRNA expression, but levels of IL-6 mRNA were increased in only two stocks, except DBA/2A (Fig. 3). ELISA was subsequently performed to compare blood protein levels of the LPS-induced pro-inflammatory cytokines in all DBA/2 stocks. Levels of TNF-α and IL-6 proteins were found to be dose-dependently increased in all LPS-treated groups, as compared to the respective Vehicle groups. Moreover, protein levels were found to be similar in all three DBA/2 stocks (Fig. 4). Overall, these results indicate that the production of septic inflammatory cytokines exhibited by the DBA/2Korl stock to LPS exposure was similar to responses of the two commercial stocks.

**Fig. 5:**
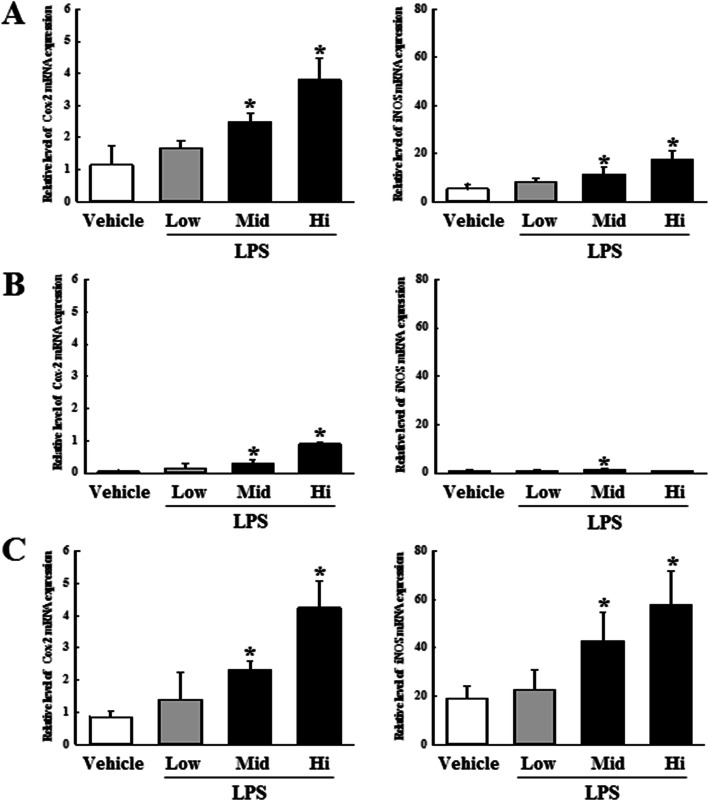
Difference in expressions of inflammatory factors in LPS treated spleen tissues harvested from the three DBA/2 stocks. The mRNA levels of inflammation related proteins (COX-2 and iNOS) were evaluated among DBA/2Korl (**a**), DBA/2A (**b**), and DBA/2B (**c**) mice after exposure to indicated concentration of LPS or Vehicle. Left panel present mRNA level of COX-2 after exposure to indicated concentration of LPS or Vehicle. Right panel present mRNA level of iNOS after exposure to indicated concentration of LPS or vehicle. *P<0.05 compared to the Vehicle treated group

Numerous studies have reported that levels of proinflammatory cytokines induced by sepsis are amplified by inflammatory factors such as COX-2 and iNOS. We therefore investigated alterations in the expressions of inflammatory factors in the three DBA/2 stocks. After LPS stimulation at various concentrations for 20 h, the gene expressions of COX-2 and iNOS in spleen cells obtained from each DBA/2 stock were observed in RT-PCR. LPS administration increased expressions of COX-2 and iNOS mRNA in a dose-dependent manner. However, although the iNOS expression of DBA/2A was only slightly altered, the other stocks showed similar COX-2 and iNOS expressions (Fig. 5).

## Discussion

There exists some ambiguity in the use of experimental animal models for the study of sepsis. The human response to sepsis and the experimental animal model at the genomic level show significant differences. In particular, several studies report that rodent animal sepsis models are inappropriate for comparing syngeneic rodent models with a subject having complex characteristics, such as humans, when considering allogenic endotoxin-induced septic models [13, 15, 24, 25]. Despite these reports, application of a sepsis model using mice is useful for studying human sepsis because various functions of a mouse experimental animal model based on biological similarity and pathophysiology of human sepsis are possible. For example, the mortality model that causes rapid death can be compared with human sepsis patients who have a low probability of death that is more likely to occur within a few days rather than hours after the outbreak of disease. Pretreatment experiments for sepsis in a mouse animal model can therefore help to understand the treatment of human patients [26, 27]. In the current study, DBA/2 mice are used to provide basic knowledge for correct selection of a mouse species to induce animal models that would help improve understanding of human sepsis more than studies using mouse models with human-like sepsis phenotypes.

LPS is a single component of the complex pathogen-related molecular pattern (PAMP) released by Gram-negative organisms [27]. The sepsis model using this LPS is generally constructed by intraperitoneal injection of LPS into the mouse, and has been used in numerous studies since reporting the similarity of this model with the pathophysiology of severe human sepsis [28]. However, the limitations of mouse sepsis using LPS is the inabilty to explain sepsis for gram-positive microorganisms, the tendency of inflammatory cytokines to appear and disappear more rapidly when compared to human sepsis, and the tendency of sepsis to depend on LPS concentration [29–33]. Despite these limitations, our current study compared three DBA/2 stocks in the sepsis model using LPS (which is not only a commonly used model as mentioned above), but also LPS is a specific toll-like receptor (TLR) pathway such as TLR4. The LPS model has well-documented biological mechanisms and pathways, such as the immune response, accumulated through results of many studies. Therefore, we applied this model for comparative analysis of the sepsis reaction between DBA/2 stocks, since it provides useful analysis index for comparison between stocks.

In general, mortality from sepsis is about 30%, with severe sepsis observed in up to 50%, and sepsis shock in 80% patients [34]. Therefore, one of the final goals of the clinical approach to sepsis is to reduce the mortality rate. We applied LPS as low (2 mg/kg), medium (5 mg/kg) and high (10 mg/kg) concentrations to investigate mouse survival and sepsis-related responses within a day. Several studies have reported that C57BL/6 exhibits a survival rate of 25% within approximately 40 h at 20 mg/kg, whereas the DBA/2 mouse is more responsive to LPS concentration than the C57BL/6 mouse [20, 35–37]. Comparison of the survival rate (Fig. 1) in the three stocks revealed that DBA/2A stock has a slightly better reactivity to LPS, and the survival rate was decreased at a high concentration at 20 h. However, there was no significant difference among the three DBA/2 stocks. It is well known that influencing the survival rate from LPS-induced sepsis leads to death due to tissue damage and multiple organ failure caused by excessive immune responses [38, 39]. As shown in Fig. 2, no significant abnormalities were detected in the liver and spleen tissues due to LPS treatment in the three DBA/2 stocks. These results support that the survival rates of the DBA/2 stocks are not significantly different.

Apart from septic inflammatory reactions, macrophages also play an important role in general inflammatory reactions. LPS binds to the TLR4 receptor, which is also expressed on a variety of immune cells but is especially abundant in macrophages, providing evidence that macrophages are mainly involved in the LPS-induced inflammatory reactions. Macrophages play an important role in the secretion of inflammatory cytokines such as TNF-α and IL-6, host defense against infection, and recovery of damaged tissues [17, 31]. Therefore, inhibiting the overproduction of inflammatory cytokines in immune cells, including macrophages, is one of the treatments for suppressing inflammatory diseases [40]. In the current study, the direct distribution analysis of macrophages was not performed, but the expression of inflammatory cytokines in mouse whole blood were compared in the three DBA/2 stocks. Figures 3 and 4 shows that the expression and secretion of inflammatory cytokines in DBA/2 stocks is dependent on the concentration of LPS in the LPS-induced sepsis model, with no variations among the three stocks. An analysis of macrophage functions in LPS-induced sepsis will be identified for future studies. Next, we analyzed the inflammation factors related to amplification of sepsis by inflammatory cytokines. It is well known that NO is produced in the inflammatory response, after which the peroxide (ONOO^−^) is synthesized. Subsequently, large amounts of cytotoxic ONOO^−^ lead to tissue damage due to oxidative stress and DNA damage [41]. Moreover, another inflammatory factor, COX-2, is a key component for synthesizing prostaglandin (PG), thereby mediating inflammatory reactions that cause fever, pain, hypersensitivity and edema [42]. Figure 5 shows the expressions of iNOS and COX-2 mRNA in the DBA/2 mouse after exposure to varying LPS concentrations. Both iNOS and COX-2 genes show dose-dependent increased expressions, and this pattern was observed in all three DBA/2 stocks, with no significant difference.

## Conclusion

In conclusion, we compared the inflammatory response of animal models for sepsis by administering LPS in three DBA/2 stocks. We examined the differences of survival rate and organ failure in LPS-induced sepsis of animal models among the stocks, namely, DBA/2Korl, DBA/2A, and DBA/2B. Moreover, we found that production of inflammatory cytokines and related factors, and activation of transcriptional factors after LPS exposure, were similar for all three DBA/2 stocks. Our results suggest that DBA/2 mice from other commercial suppliers, as well as the DBA/2Korl mice, can be extensively applied to produce LPS-induced sepsis animal models.

## Data Availability

Available.

## Abbreviations

AAALAC: Association for Assessment and Accreditation of Laboratory Animal Care
CLP: Cecum Ligation and Puncture
FDA: Food and Drug Administration
H&E: Hematoxylin and Eosin
LPS: Lipopolysaccharide
SDS-PAGE: Sodium dodecyl sulfate-polyacrylamide gel electrophoresis
SPF: Specific Pathogen-free
TLR: Toll-like receptor
PG: Prostaglandin
